# Multimodal Fusion-Driven Pesticide Residue Detection: Principles, Applications, and Emerging Trends

**DOI:** 10.3390/nano15171305

**Published:** 2025-08-24

**Authors:** Mei Wang, Zhenchang Liu, Fulin Yang, Quan Bu, Xianghai Song, Shouqi Yuan

**Affiliations:** 1 School of Agricultural Engineering, Jiangsu University, Zhenjiang 212013, China; 2222316077@stmail.ujs.edu.cn (Z.L.); qbu@ujs.edu.cn (Q.B.); 2 Institute of the Green Chemistry and Chemical Technology, School of Chemistry and Chemical Engineering, Jiangsu University, Zhenjiang 212013, China; 3 Research Center of Fluid Machinery Engineering and Technology, Jiangsu University, Zhenjiang 212013, China

**Keywords:** crop safety, spectral analysis technology, biosensing technology, nanotechnology, pesticide residue

## Abstract

Pesticides are essential for modern agriculture but leave harmful residues that threaten human health and ecosystems. This paper reviews key pesticide detection technologies, including chromatography and mass spectrometry, spectroscopic methods, biosensing (aptamer/enzyme sensors), and emerging technologies (nanomaterials, AI). Chromatography-mass spectrometry remains the gold standard for lab-based precision, while spectroscopic techniques enable non-destructive, multi-component analysis. Biosensors offer portable, real-time field detection with high specificity. Emerging innovations, such as nano-enhanced sensors and AI-driven data analysis, are improving sensitivity and efficiency. Despite progress, challenges persist in sensitivity, cost, and operational complexity. Future research should focus on biomimetic materials for specificity, femtogram-level nano-enhanced detection, microfluidic “sample-to-result” systems, and cost-effective smart manufacturing. Addressing these gaps will strengthen food safety from farm to table while protecting ecological balance. This overview aids researchers in method selection, supports regulatory optimization, and evaluates sustainable pest control strategies.

## 1. Introduction

Pesticides are indispensable in modern agriculture, significantly boosting crop yields by controlling pests and diseases, thereby ensuring global food supplies. However, excessive pesticide use has led to increasingly prominent residue issues [1,2]. From a public health perspective, these residues can bioaccumulate through the food chain, posing serious health risks such as chronic toxicity, immune suppression, and potential carcinogenic effects in humans. These risks are particularly heightened for infant development. Environmentally, pesticide residues contaminate soil and water bodies, disrupt ecological balance, reduce biodiversity, and can cause long-term pollution through groundwater infiltration [3,4]. Consequently, while maintaining agricultural productivity remains essential, there is an urgent need to regulate pesticide application more strictly and to promote sustainable, eco-friendly pest control technologies. Establishing a robust pesticide residue detection system is crucial for advancing green control technologies. Precise and efficient detection methods not only monitor agricultural product safety but also provide a scientific basis for evaluating green control strategies.

The evolution of pesticide detection technology can be traced back to the mid-20th century, marking the beginning of systematic efforts to monitor chemical residues in agricultural and environmental systems. Early methods relied primarily on chemical analysis (e.g., colorimetry, titration) and chromatographic separation techniques (e.g., gas chromatography, liquid chromatography), whose accuracy was limited by instrument sensitivity and sample preparation complexity. After the 1980s, breakthroughs in immunoassay led to the widespread adoption of rapid detection methods like enzyme-linked immunosorbent assays and colloidal gold test strips, enabling on-site screening and semi-quantitative analysis. Entering the 21st century, hyphenated mass spectrometry (MS) techniques (e.g., GC-MS, LC-MS), offering high sensitivity and multi-residue detection capabilities, became the laboratory “gold standard” [5]. In recent years, the integration of cross-disciplinary technologies such as nanomaterials, biosensors, and CRISPR gene editing has driven detection towards miniaturization, intelligence, and real-time capability [6,7].

The field of pesticide detection is currently undergoing a transformative phase, driven by the convergence of advanced technologies and an increasing emphasis on precision in agricultural and food safety management. On one hand, the combination of novel materials and artificial intelligence algorithms significantly enhances detection throughput and data analysis efficiency. On the other hand, increasingly stringent global Maximum Residue Limits (MRLs) are pushing detection technologies to continually break sensitivity barriers [8,9]. However, challenges such as interference from complex matrices (e.g., tea, honey), simultaneous multi-component identification, equipment cost, and standardization still hinder large-scale application. Looking ahead, as disruptive technologies like microfluidic chips and in situ MS imaging mature, pesticide detection is poised for systemic innovation in sensitivity, specificity, and practicality, establishing a more robust technical safeguard for food and ecological security.

This article presents a systematic classification framework for pesticide residue detection technologies, designed to offer readers a comprehensive and structured understanding of the current technological landscape. Based on detection principles and application scenarios, existing technologies are primarily categorized into major groups: Chromatography and MS, Spectroscopic Methods, Biosensing Technologies, and Emerging Technologies. These correspond to the needs for laboratory precision testing, on-site rapid screening, and intelligent real-time monitoring, respectively [5]. By comparing core parameters like detection limits, throughput, and cost across these technologies, this paper seeks to reveal the underlying logic and synergistic relationships driving technological advancement. This analysis will provide researchers with references for technology selection, assist regulatory bodies in optimizing detection resource allocation, and offer methodological support for evaluating the effectiveness of green control technologies [3,6], thereby demonstrating significant theoretical and practical value.

## 2. Classification of Pesticide Detection Technologies

### 2.1. Chromatography-Mass Spectrometry

Chromatography serves as a vital technique for pesticide separation and analysis, leveraging differences in partition coefficients between stationary and mobile phases to resolve components. When coupled with detection systems such as diode array detectors (DAD), chromatography achieves reliable sensitivity at the parts-per-million (ppm) level, even in challenging matrices like fruits and vegetables [10]. MS utilizes molecular ionization and mass analysis to deliver precise molecular weights and elucidate molecular structures via fragment ion spectra—crucial for identifying emerging pesticide metabolites. Hyphenated GC-MS and LC-MS systems synergistically combine chromatographic separation with MS structural characterization, effectively overcoming traditional limitations like high false positives and poor matrix interference resistance [11,12].

Recent years have witnessed significant advances in chromatography-mass spectrometry (GC-MS) hyphenated techniques for detecting pesticide mixtures. For instance, an analytical method combining QuEChERS (Quick, Easy, Cheap, Effective, Rugged, Safe, a rapid sample pretreatment technique for agricultural product analysis) with UHPLC-MS/MS was developed to simultaneously detect pesticide and veterinary drug residues in agricultural soil. Using an acetonitrile-acetic acid (99:1) extraction system and C18 chromatographic separation, this approach achieved efficient separation of 68 target compounds within 14 min. The implementation of dynamic Multiple Reaction Monitoring in MS significantly enhanced detection sensitivity (LOQ: 0.01–0.5 μg/kg), while dual confirmation through characteristic ion pairs and retention times ensured result accuracy. This chromatographic-spectrometric synergy effectively overcame soil matrix interference, providing a reliable technical solution for environmental monitoring [13].

In a comprehensive study on indoxacarb residue dynamics in radishes, Zhao et al. applied isotope-labeled internal standard-corrected ultra-high-performance liquid chromatography–tandem mass spectrometry (UHPLC-MS/MS), achieving a highly sensitive quantitation limit of 0.005 mg/kg. The research revealed significant tissue-specific differences: a half-life of 2.6–6.8 days in leaves versus 3.3–8.0 days in roots, with terminal residues reaching up to 25.46 mg/kg in leaves but remaining below 0.3 mg/kg in roots. Monte Carlo simulation-based risk assessment indicated a dietary risk quotient exceeding 100%, providing scientific justification for establishing differentiated MRLs [14].

**Figure 1:**
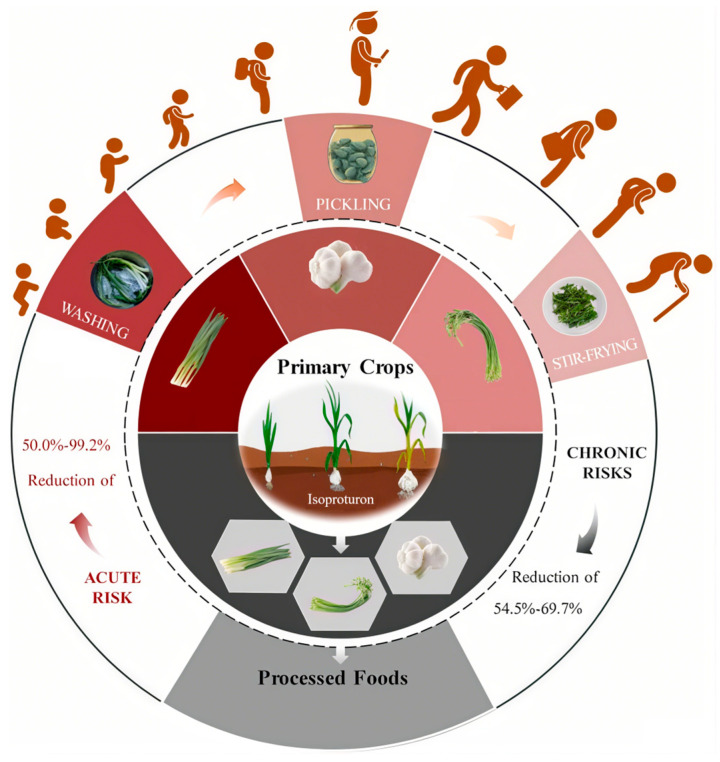
UHPLC-MS detection of IPU during the entire process of garlic cultivation and processing [15].

Chen et al. developed a highly efficient and accurate analytical method for monitoring isoproturon (IPU) residues across the entire garlic production chain—from cultivation to post-harvest processing—using ultra-high-performance liquid chromatography–tandem mass spectrometry (UHPLC-MS/MS) (Figure 1). Their method demonstrated high sensitivity (detection limits down to μg/kg levels), rapid separation (<10 min per sample), and robust matrix interference resistance, ensuring precise IPU quantification in complex matrices. Through optimized pretreatment and Multiple Reaction Monitoring, this approach effectively tracked IPU’s migration from cultivation to consumption, delivering scientific support for establishing maximum residue levels (MRLs) and assessing dietary risks. Its efficiency and precision underscore its critical value in contemporary pesticide residue analysis [15].

Pan et al. introduced a pioneering dual-mode analytical platform that integrates GC-MS with surface-enhanced Raman spectroscopy (SERS), establishing a synergistic framework for comprehensive pesticide residue analysis [16]. Optimization of the QuEChERS pre-treatment method, specifically the acetonitrile extraction and Carb/NH_2_ column purification steps, enabled GC-MS detection limits of 0.002–0.045 mg/kg for organophosphorus pesticides in vegetables and edible oils. Concurrently, the team developed a SERS aptamer sensor featuring bimetallic nanostar structures and 4-mercaptobenzonitrile labeling technology, achieving ultra-high sensitivity towards chlorpyrifos with a detection limit of 220.35 pg/mL. This dual-mode analytical system, combining “laboratory-grade precision with on-site rapid screening,” provides an innovative solution for agricultural product safety monitoring [16].

GC-MS techniques continue to advance as essential tools for pesticide residue analysis. GC-MS maintains dominance in detecting volatile pesticides, while LC-MS remains indispensable for analyzing polar and thermally labile compounds. Continuous improvements in separation conditions, detection parameters, and sample preparation techniques have significantly enhanced multi-residue screening capabilities. The adoption of High-Resolution MS has further expanded non-targeted screening applications. Moreover, the strategic integration of complementary detection technologies offers robust technical support for developing comprehensive pesticide monitoring frameworks.

### 2.2. Spectral Analysis Techniques

Spectroscopic analysis techniques primarily encompass hyperspectral imaging, SERS, and polarization spectroscopy coupled with fluorescence techniques. Hyperspectral imaging visualizes the spatial distribution of pesticide residues through continuous narrow-band imaging, offering both non-destructive analysis and multi-component detection capabilities. SERS utilizes nanostructures to amplify Raman signals, delivering parts-per-billion (ppb) level sensitivity while providing molecular fingerprints that significantly streamline sample preparation workflows. Polarization spectroscopy analyzes material structures through light polarization characteristics, and fluorescence techniques leverage light emission properties upon excitation, their integration enhances detection sensitivity and specificity.

In the field of pesticide residue detection, hyperspectral imaging, SERS, and polarization-fluorescence techniques offer distinct yet highly complementary capabilities, each addressing different analytical challenges across spatial, sensitivity, and operational domains. Hyperspectral imaging excels in non-invasive, large-area surface monitoring, enabling rapid spatial mapping of pesticide residues on agricultural commodities such as fruits and vegetables—making it particularly valuable for field-deployable, high-throughput screening. In contrast, SERS provides exceptional molecular selectivity and ultra-trace sensitivity, allowing for the identification of low-concentration residues down to the single-molecule level, which is critical for confirming contamination in complex matrices. Meanwhile, the polarization-fluorescence dual-modality approach combines high sensitivity with excellent specificity and non-destructive operation, enabling precise residue identification without sample degradation—ideal for repeated measurements and quality-sensitive applications. When integrated, these three methodologies form a synergistic analytical framework that bridges the gap between rapid field screening and confirmatory laboratory analysis. This end-to-end solution spans the entire analytical workflow—from on-site surveillance to lab-based validation—delivering unprecedented improvements in sensitivity, precision, and operational efficiency in pesticide residue monitoring [17,18,19].

#### 2.2.1. Hyperspectral Imaging Technology

##### Principle

Hyperspectral imaging systems simultaneously capture spatial distribution characteristics and continuous narrow-band spectral information of targets, generating three-dimensional data matrices comprising hundreds of spectral dimensions. The technology leverages the distinctive spectral response signatures unique to different substances. Through spectral separation devices and chemometric modeling methods, dedicated spectral identification models for pesticide residues are established. This technology has completed its transition from laboratory research to practical applications. Initially applied for qualitative discrimination of pesticide residues on fruits and vegetables (as seen in the identification of chlorpyrifos residues within the 900–1700 nm spectral range), it achieved ppm sensitivity levels [20]. Recent advancements integrate deep neural networks with multispectral co-analysis techniques, effectively mitigating interference from complex matrices such as fruit wax layers and natural pigments. This enables simultaneous quantitative analysis of multiple pesticide residues. The development of miniaturized devices further facilitates field-deployable applications, exemplified by mobile smart terminal-based instant detection systems. Current research focuses on enhancing ultra-high sensitivity, robust anti-interference capability, and intelligent processing for next-generation systems.

##### Hyperspectral Imaging Technology in Agricultural Product Safety Testing

Recent breakthroughs in hyperspectral imaging technology have opened new avenues for non-destructive and rapid inspection of agricultural product safety, particularly in the detection of surface pesticide residues. Regarding detection methodology, the innovative spectral analysis technique developed by Ma et al. combined with wavelet feature extraction has effectively addressed long-standing technical challenges including leaf deformation and autofluorescence interference [21]. This advancement laid critical groundwork for subsequent detection systems.

**Figure 2:**
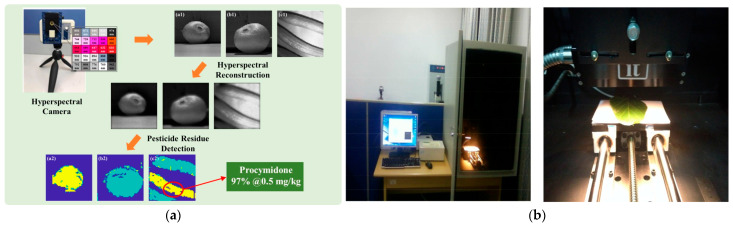
(**a**) Portable filter array hyperspectral imaging for rapid detection of pesticide residues [21]; (**b**) Vis-NIR hyperspectral imaging system for acquiring images from mulberries [22].

Building directly on this progress, researchers have developed a series of advanced detection platforms, each tailored to specific operational and analytical needs. For instance, a portable multispectral system (450–850 nm) demonstrated a chlorpyrifos detection limit of 0.2 mg/kg, representing 50% higher sensitivity than China’s regulatory standard (Figure 2a) [21]; A near-infrared imaging system (900–1700 nm) achieved quantitative analysis at 0.3 mg/kg levels while simultaneously visualizing pesticide residue spatial distribution (Figure 2b) [22]; A multispectral fusion platform (400–1000 nm) employed feature selection algorithms to attain R^2^ = 0.92 prediction accuracy with support vector regression modeling [23]. The establishment of these detection systems signifies a new stage in agricultural product safety inspection technology.

Building upon these technological advances, researchers have conducted multiple representative application studies. Among these, the work by Zhou Sun et al. stands out as a particularly innovative example [24]. By synergistically integrating the molecular structural characteristics of pesticides with wavelet transform-based spectral analysis, the team developed a novel, rapid identification method for pesticide residues in lettuce. The near-infrared hyperspectral imaging system (870–1780 nm) was utilized to acquire the near-infrared hyperspectral image information of five different pesticide residues (dimethoate, acephate, phoxim, dichlorvos, and avermectin) on lettuce surfaces. The ENVI software was employed to select regions of interest (ROI) from the sample hyperspectral images to obtain near-infrared spectral data. Furthermore, a method combining Chemical Molecular Structure with Wavelet Transform (CMS-WT) was applied for extracting the most influential wavelengths. By comparing the chemical molecular structures of the four pesticides, four characteristic intervals were identified. Based on the extracted characteristic spectral data, an SVM model was constructed. Results demonstrated that the calibration and prediction accuracy of the SVM model established using the optimal eight-feature combination both reached 100%. This confirms the feasibility and effectiveness of the CMS-WT feature extraction algorithm in constructing models for different pesticide residues in lettuce.

Moreover, based on hyperspectral technology combined with chlorophyll fluorescence spectroscopy, Sun et al. developed a method integrating wavelet transform and the MD-MCCV algorithm (WT-MD-MCCV) to identify the optimal wavelengths from spectral data. Hyperspectral and chlorophyll fluorescence spectral data of lettuce leaf samples under five different pesticide residue concentrations were acquired using a hyperspectral data acquisition device and a Cary Eclipse fluorescence spectrophotometer. Support Vector Regression (SVR) was then employed to construct prediction models. The results indicated that the WT-MD-MCCV algorithm, when applied to the combined hyperspectral data, chlorophyll fluorescence spectral data, and the fused hyperspectral-chlorophyll fluorescence data, performed best among the nine SVR models, demonstrating that hyperspectral technology combined with chlorophyll fluorescence spectroscopy can be effectively used to identify pesticide residue levels in lettuce leaves [25]. These breakthrough not only resolves spectral overlap issues inherent in conventional methods but also delivers efficient technical support for agricultural product safety regulation.

#### 2.2.2. SERS Technology

##### Principles and Classification

SERS leverages the localized surface plasmon resonance effect of noble metal nanostructures (e.g., gold/silver substrates) to generate substantial electromagnetic field enhancement, amplifying the Raman signal intensity of adsorbed molecules by 10^6^–10^8^ times [26]. When pesticide molecules form chemical bonds with the metal surface, the resultant alteration of molecular electron cloud distribution induces additional chemical enhancement, contributing a further 10- to 100-fold signal amplification [27]. The synergistic action of these enhancement mechanisms enables detection sensitivity reaching levels of 0.1 ppb. This technique establishes molecular fingerprint spectra by identifying characteristic vibrational peaks (e.g., the P=O bond peak at 750 cm^−1^ for organophosphorus compounds), allowing efficient screening of multiple pesticide residues in complex samples through integration with chemometric methods [28].

SERS has emerged as a cornerstone technique in the detection of pesticide residues, owing to its exceptional sensitivity and molecular specificity. Its widespread adoption is driven by three key operational advantages: (i) minimal sample preparation, enabling direct analysis of liquid samples; (ii) rapid measurement kinetics, with individual analyses typically completed within 15 min; and (iii) inherent capacity for multiplex detection of multiple pesticide residues in a single measurement [29]. To further enhance performance, researchers have employed advanced strategies such as functionalizing substrates with metal–organic frameworks (MOFs) and designing target-specific recognition probes (e.g., aptamers, molecularly imprinted polymers). Nevertheless, practical deployment of SERS in real-world settings remains constrained by persistent challenges, including matrix interference from complex agricultural samples and the lack of standardized protocols for substrate fabrication, calibration, and data interpretation [30].

In response to these demands, significant progress has been made in substrate engineering and system integration, leading to the development of four distinct classes of SERS platforms—each designed to address specific analytical requirements.

(1) Flexible smart substrates utilize environmentally responsive materials (e.g., polyacrylamide hydrogels) to dynamically modulate nanoparticle spacing through solvent-induced deformation. This significantly enhances hot-spot density, making them particularly suitable for irregular surface detection [30,31].
(2) Rigid nanostructured substrates (e.g., dendritic AgNPs, Au@Ag core–shell structures) optimize electromagnetic field distribution through precise morphological control (e.g., dendritic architecture, core–shell thickness). This approach elevates detection sensitivity by three orders of magnitude [32,33,34].
(3) Penetrative functionalized substrates (e.g., Ag/HA/PVA microneedles) utilize microneedle arrays to overcome conventional surface analysis limitations, enabling in situ detection of deep-layer substances [35,36].
(4) Porous adsorptive substrates (e.g., activated carbon-supported AC@AgNPs, mesoporous scaffold-based AuNPs/MSN) leverage the enrichment capability of high-surface-area materials (e.g., mesoporous silica) combined with plasmonic effects. This synergy achieves detection limits as low as 0.01 ppb for certain organophosphorus pesticides [28,37].

##### SERS in Agricultural Product Safety Testing

SERS has emerged as a pivotal analytical tool for pesticide residue detection, leveraging its unique dual electromagnetic and chemical enhancement mechanisms [38,39]. The technique’s most significant advantages include: (1) Ultra-high sensitivity at the ppb level; (2) Rapid detection capability within 15 min; (3) Simultaneous multi-component analysis functionality. Driven by rapid advances in nanomaterials science, researchers have recently overcome critical technical barriers, such as substrate-curvature compatibility and deep-layer detection, through innovative designs including flexible smart substrates and core–shell nanostructures. Particularly for complex matrices like tea, SERS demonstrates exceptional detection performance surpassing international standards by two orders of magnitude.

At the implementation level, breakthroughs in SERS technology primarily manifest across three dimensions. Firstly, through structural engineering approaches including nanoparticle morphology control and ordered mesoporous supports, achieving substantial signal amplification [40]. Then, enabling curved-surface sample analysis and simultaneous multicomponent detection [41]. Finally, development permitting field-based rapid screening. These innovations collectively enhance methodological robustness, demonstrating good reproducibility and acceptable recovery rates, while significantly improving interference resistance [42,43].

**Figure 3:**
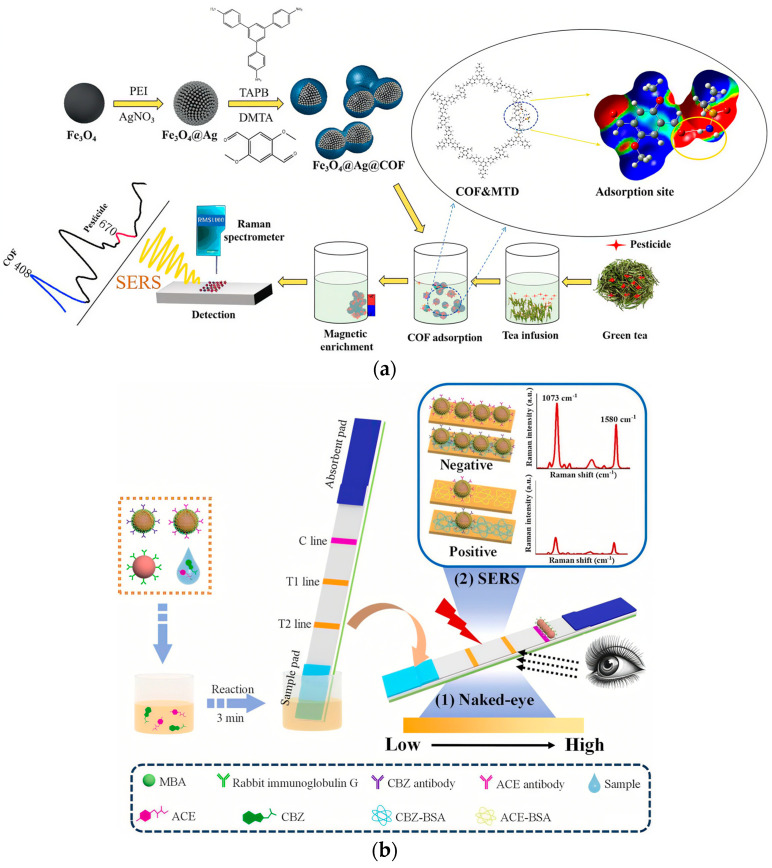
(**a**) An efficient molecular enrichment and magnetic separation flow with Fe_3_O_4_@Ag@COF for ratiometric SERS detection of methamidophos [46]; (**b**) rapid dual-mode immunoassay based on SERS for detecting two types of pesticides in fruits [49].

SERS technology has achieved remarkable progress in the detection of pesticide residues in tea. For instance, three-dimensionally self-assembled Au-Ag core–shell supraspheres generate high-density signal hotspots, enhancing detection sensitivity by 8 times compared to traditional substrates [44]. Zhu et al. developed a rapid, low-cost, and highly sensitive method for qualitative and quantitative analysis of chlorpyrifos residues in tea by synthesizing gold-core silver-shell nanoparticles (Au@Ag NPs) with high enhancement factors as SERS substrates and combining them with chemometric algorithms for spectral analysis. The classification accuracy for tea samples reached 90.84–100% [45]. Moreover, Zhang et al.’s designed multifunctional Fe_3_O_4_@Ag@COF nanocomposite (Figure 3a) ingeniously combines sample enrichment and internal standard calibration functions, demonstrating a broad linear detection range of 10^−4^ M to 10^−9^ M for methamidophos with an exceptionally low detection limit of 8.3 × 10^−5^ mg/kg, surpassing international standards by two orders of magnitude [46]. By combining this technique with chemometric analysis methods and magnetic separation technology, it effectively overcomes interference from the complex tea matrix. The detection results demonstrated high consistency with GC-MS), with errors less than 7%.

Based on innovative breakthroughs in substrate materials, SERS demonstrates unique advantages in addressing the increasingly complex challenges of mixed pesticide residues in modern agriculture. Research indicates that coexisting pesticide residues may amplify toxicity risks, creating an urgent need for innovative detection solutions.

To address this critical challenge, researchers have developed multiple innovative approaches. Li et al. [47] engineered a sophisticated solution, titanium dioxide/silver composite structures that not only exhibit superior detection sensitivity but also incorporate self-cleaning properties, making them particularly effective for analyzing complex environmental samples. For instance, it can be used for rapid detection of pymetrozine and thiram in tea samples. Kim et al. [48] pushed the boundaries of detection efficiency by integrating PCA-SVM algorithms into an intelligent analysis system that achieves >90% accuracy while reducing detection time to merely 3 min.

Advancing this technological progression, Guo et al. [49] pioneered an approach (Figure 3b) utilizing gold nanoparticle-antibody probes that effectively combine immunochromatographic screening with SERS quantification, enabling simultaneous detection of acetamiprid and carbendazim two pesticides within just 15 min. Through the work of Hasi et al. [50], the field witnessed further innovation with their ingenious integration of thin-layer chromatography and SERS, achieving rapid separation and identification of thiram, triadimefon, benzimidazole, and thiamethoxam four pesticide mixtures in a record 5 min timeframe.

Collectively, these cumulative advancements establish a comprehensive technological framework for pesticide detection, systematically addressing challenges through probe design optimization, substrate enhancement, and intelligent algorithm integration. With demonstrated reliability and growing potential for practical implementation, SERS technology is poised to revolutionize food safety monitoring, particularly as AI integration and portable device development continue to advance.

#### 2.2.3. Polarization Spectroscopy and Fluorescence Technology

Polarization spectroscopy derives molecular structural information by analyzing matter’s interaction with polarized light, wherein molecular anisotropy alters the light’s polarization state, changes directly correlated with molecular orientation and structure. By detecting differential absorption, scattering, or emission of polarized light, characteristic spectra are obtained, making this technique particularly suitable for analytical studies distinguishing isotropic and anisotropic molecular systems. Specifically applied to pesticide detection, polarization spectroscopy quantifies polarization state changes following light-sample interactions to identify target molecules, demonstrating exceptional sensitivity for chiral organophosphorus compounds (e.g., chlorpyrifos). These compounds induce characteristic polarization angle shifts exhibiting excellent linear concentration dependence (R^2^ > 0.98) [51]. Complementarily, fluorescence technology leverages molecular stimulated emission principles: Pyrethroid pesticides excited by 280 nm ultraviolet radiation exhibit specific fluorescence emission peaks at 420 nm, with intensity directly proportional to residue levels [52]. Recent breakthroughs include integrating excitation-emission matrices with MEA-BP neural networks to achieve 1.325% detection error for carbamates [53]; developing fluorescence spectroscopy-PLS multivariate calibration models enabling synchronous quantification of zhongshengmycin, paclobutrazol, boscalid, and pyridaben four pesticides (R^2^ > 0.98) [54]; and establishing a high-efficiency residue removal protocol via sodium bicarbonate solution immersion (>90% removal within 12 min), collectively forming an integrated workflow from precise detection to practical decontamination.

Fluorescence Polarization Immunoassay (FPIA) technology establishes a novel paradigm for pesticide residue detection by integrating the advantages of two optical methodologies. Its core mechanism relies on the increase in polarization degree resulting from reduced molecular rotation when fluorescently labeled antigens bind to antibodies, a parameter inversely correlated with analyte concentration. Addressing the limitation of conventional fluorescence methods, which are susceptible to chlorophyll interference, FPIA leverages the unique benefits of polarization signals to achieve sub-ppb detection limits for organophosphorus pesticides with a 3- to 5-fold signal-to-noise enhancement [55]. The incorporation of quantum dot labeling significantly improves signal stability, while integration with microfluidic chips reduces analysis time to under 8 min, substantially enhancing field-testing efficiency.

**Figure 4:**
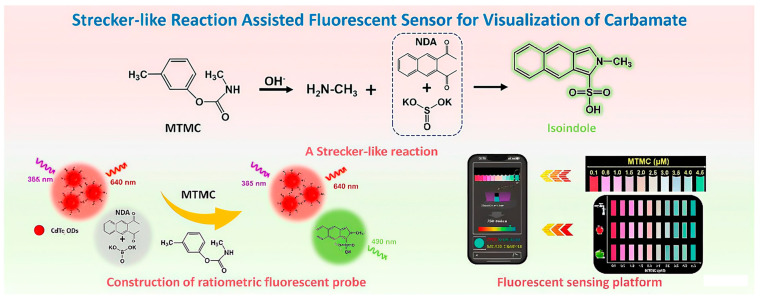
Strecker-like Reaction Assisted Fluorescent Sensor for Visualization of Carbamate [56].

Jiang et al. achieved a breakthrough in rapid detection technology by developing a ratiometric fluorescent nanoprobe system that provides an innovative solution for glyphosate detection. This technology leverages the fluorescence resonance energy transfer effect between blue carbon dots (CDs) and gold nanoclusters (Au NCs), enabling ultrasensitive detection at 4.19 nM within 2 s (Figure 4) [56]. Furthermore, a breakthrough was achieved by integrating a smartphone color recognizer with a 3D-printed portable device, establishing an enzyme-free visual detection platform. This platform utilizes a composite Rhodamine B-Ag@Au nanoprobe to enable visual and semi-quantitative detection of organophosphorus pesticide residues, successfully addressing the technical bottleneck of real-time monitoring of organophosphorus pesticides in the field [57]. This integrated design maintains laboratory-level accuracy while enabling simplified field operation, providing a practical tool for agricultural product safety supervision.

Polarization spectroscopy and fluorescence-based methods have emerged as complementary analytical approaches for pesticide residue detection, each offering distinct advantages. Polarization spectroscopy achieves specific identification by analyzing polarization state changes induced by chiral pesticides, while fluorescence quantification relies on characteristic emission peaks. FPIA synergizes these strengths to achieve ppb-level detection limits. Quantum dot labeling and microfluidic technology further reduce analysis time to 8 min. Ratiometric fluorescent probes (CDs/Au NCs) enable ultrasensitive detection at 4.19 nM, and smartphone-based platforms have propelled field-deployable rapid testing applications.

However, spectroscopic techniques still face significant limitations in pesticide detection, particularly for low-concentration (<1 nM) and multi-target analysis [49]. Conventional methods such as UV-Vis spectroscopy lack sufficient sensitivity and require signal enhancement using precious metal nanomaterials (e.g., Au@Ag core–shell structures), which are costly and complex to prepare [57]. In multi-target detection, the binding between organophosphorus pesticides (OPs) and metal nanoparticles is susceptible to interference from thiol compounds [57], while overlapping fluorescence peaks of multi-component pesticides (e.g., λ-cyhalothrin) necessitate complex deconvolution algorithms [56]. Matrix interferences in real samples (e.g., apples, cabbage) can quench fluorescence signals, leading to recovery variations of up to 8% even with internal standard calibration [55]. Current techniques (e.g., FRET/IFE) require customized probes for different pesticides, making universal multi-residue detection challenging [52]. Researchers have developed multifunctional nanocomposites (e.g., microneedle SERS arrays) [35] and intelligent algorithm-assisted spectral analysis, but challenges remain in instrument miniaturization and cost-effectiveness. Therefore, novel high-efficiency detection technologies are still needed for low-concentration, multi-target pesticide analysis.

### 2.3. Biosensing Technology

Biosensing technologies for pesticide detection are broadly classified into two major categories based on their molecular recognition elements: aptamer-based sensors and enzyme-based sensors [58,59]. Aptamer sensors utilize the specific binding between nucleic acid aptamers and target molecules to generate measurable signal changes, offering high affinity and programmable design flexibility. Enzyme sensors operate on the principle of pesticide-induced inhibition of specific enzyme activity (e.g., cholinesterase), achieving detection through enzymatic reaction signal transduction [60]. These technologies achieve detection sensitivities at the ppb level, representing a 1–2 order of magnitude improvement over conventional methods, with analysis times of merely 10–15 min. Representative applications include: gold nanoparticle colorimetric aptamer sensors for organophosphorus pesticide detection, and acetylcholinesterase-modified electrodes for carbamate pesticide analysis. Their portable designs enable real-time field monitoring, while nanomaterial-enhanced interference resistance provides a highly efficient solution for pesticide residue supervision.

#### 2.3.1. Adaptive Sensor

Aptamer-based sensors represent a class of molecular recognition devices centered on nucleic acid aptamers. Their core operational principle relies on detectable signals generated through the specific binding between aptamers and target molecules, including proteins, small molecules, and cells [61,62]. Based on detection methodology, these sensors are systematically classified into three categories: optical, electrochemical, and nanomaterial-enhanced types [60,63,64]. Optical sensors encompass fluorescent aptasensors, colorimetric aptasensors, and SERS platforms, while electrochemical variants include impedimetric, amperometric, and potentiometric configurations [59]. Notably, emerging technologies such as wearable biosensors and microfluidics-integrated detection platforms are gaining significant research traction [65,66]. This technology demonstrates three key advantages: (i) Exceptional specificity, enabled by directed screening through Systematic Evolution of Ligands by Exponential Enrichment (SELEX); (ii) ultrahigh sensitivity reaching femtomolar (fM) levels, further enhanced through nanomaterial modification; and (iii) superior stability and chemical synthesis feasibility compared to traditional antibodies [67]. Current research efforts primarily focus on cutting-edge areas including microfluidic system integration, development of multiplex detection arrays, and optimization of automated screening technologies.

Building upon the aforementioned technical principles—particularly their high specificity, programmability, and signal transduction versatility—aptamer-based sensors have advanced significantly in recent years, finding growing applications in diverse fields of analytical and environmental monitoring. Notably, in the critical domain of agricultural product safety testing, aptamer sensors have demonstrated distinct advantages, including rapid response, high selectivity toward target contaminants, and compatibility with complex sample matrices.

Among the various signal transduction strategies, fluorescent aptamer sensors have emerged as a particularly powerful platform due to their high sensitivity and real-time detection capability. A representative example is the development of a luminescence resonance energy transfer (LRET)-based biosensor that integrates upconversion nanoparticles (UCNPs) as donors with MnO_2_ nanosheets as quenchers. By constructing an energy donor-acceptor pair between NaYF_4_^3+^, Tm^3+^ UCNPs and MnO_2_ nanosheets, this platform leverages carbendazim-specific aptamers to trigger MnO_2_ degradation, thereby restoring upconversion luminescence signals. This approach achieves an ultra-low detection limit of 0.02 ppb and 50 times more sensitive than conventional ELISA methods. Key innovations include: (i) Utilizing the LRET mechanism to effectively eliminate background interference inherent in traditional fluorescence detection; (ii) enabling signal-switchable responses through controllable degradation of MnO_2_ nanosheets, significantly enhancing detection specificity; and (iii) maintaining exceptional selectivity and stability in complex food matrices (e.g., apples, tomatoes). This technology synergistically integrates the high specificity of aptamers with the anti-photobleaching properties of UCNPs, offering a novel solution for on-site pesticide residue detection with superior sensitivity and interference resistance [68].

Breakthroughs in colorimetric aptamer sensors primarily stem from innovative integrations of nanomaterials with signal amplification strategies [69,70]. For example, Hu et al. developed a colorimetric chemical sensor for the simple and rapid detection of diazinon pesticide in agricultural products. This sensor primarily relies on the inhibition of the peroxidase-mimicking catalytic activity of gold nanoparticles (AuNPs). When combined with hydrogen peroxide, the nanoparticles can oxidize the substrate o-phenylenediamine (OPD), generating the final product 2,3-diaminophenazine, which exhibits a characteristic absorption peak at 450 nm and appears yellow. The sensor can detect diazinon within a linear range of 10 μg L^−1^ to 400 μg L^−1^, with a low detection limit (LOD) of 4.7 μg L^−1^, and demonstrates excellent selectivity against other competing pesticides [71]. Key technical advancements include: (i) Utilizing gold nanoparticle (AuNP) aggregation/dispersion state transitions as visual detection signals, where aptamer-target binding triggers interparticle distance changes for rapid naked-eye readouts; (ii) developing multifunctional nanozymes to couple aptamer recognition with enzymatic chromogenic amplification, enhancing sensitivity by 10–100 times; and (iii) constructing magnetic separation-colorimetric dual-mode platforms using magnetic nanoparticles for target enrichment and background interference elimination, achieving 92–107% recovery rates in real-sample analyses. These sensors universally feature operational simplicity (10–20 min procedures) and instrument-free operation, with detection limits reaching the pM range (e.g., 0.33 pM for kanamycin). Incorporation of internal standard molecules enhances reproducibility, providing robust technical support for on-site food safety screening [69].

Significant progress in SERS aptasensors for pesticide mixture detection spans three key areas. In nanostructure design, the core-internal standard-shell configuration (Au@4-MBA@Ag) employs internal standard molecules to calibrate signals, thereby reducing kanamycin’s detection limit to 0.33 pM (RSD < 8.3%) [62]. Concurrently, three-dimensional porous GO@Au nanosheets generate high-density hotspots, achieving exceptional patulin detection sensitivity at 0.01 pg/mL [72]. Regarding recognition mechanisms, the innovative integration of high-specificity aptamers with competitive strategies enables organophosphorus pesticide detection at 0.1 ppb levels. The MSN-supported ordered gold nanoparticle substrate (AuNPs@MSN) maintains >90% signal stability over 30 days, effectively resolving traditional colloidal aggregation challenges [43]. For practical applications, all studies demonstrate 92–107% recovery rates with <10% RSD, while reducing detection times to under 20 min. These portable systems exhibit strong matrix interference resistance, collectively providing sensitive, stable on-site solutions for simultaneous multi-pesticide residue detection in complex samples.

Electrochemical aptasensors exhibit transformative breakthroughs through synergistic advancements in nanomaterial interface engineering and signal amplification technologies [61,73]. For instance, Almenhali et al. developed a graphene-based electrochemical biosensor utilizing three different aptamers to detect clothianidin, thiamethoxam, and thiacloprid, respectively. Compared to previously reported aptasensors, this biosensor exhibits excellent sensitivity, with a linear detection range from 0.01 ng/mL to 100 ng/mL for clothianidin, thiamethoxam, and thiacloprid. It also demonstrates outstanding selectivity toward these three analytes [74]. Wang et al. constructed an acetylcholinesterase (AChE) sensing platform based on Pt/MoS_2_/Ti_3_C_2_ MXene (Pt/MoS_2_/TM) loaded on glassy carbon electrodes (GCEs). The three-dimensional layered structure enhances conductivity and catalytic activity, significantly improving signal response. Under optimal efficiency and sensitivity, the AChE-Chit/Pt/MoS_2_/TM/GCE sensing platform exhibits comparable analytical performance and a wide detection range for chlorpyrifos (from 10^−12^ to 10^−6^ M), with a low detection limit of 4.71 × 10^−13^ M. Furthermore, the biosensor was successfully applied to detect organophosphorus pesticides in three types of fruits and vegetables, demonstrating good feasibility and recovery rates ranging from 94.81% to 104.09% [75]. In detection mechanisms, thionine probes facilitate simultaneous quantification of neonicotinoid pesticides like imidacloprid (LOD: 0.03–0.05 ng/mL) [51], while organic photoelectrochemical transistors amplify interfacial charge transfer signals, elevating T-2 toxin sensitivity to 3.2 pg/mL [76]. Practical implementations consistently achieve 92–107% recovery rates with robust interference resistance (cross-reactivity < 8.5%). Notably, miniaturized designs such as ZnO nanorod array sensors enable portable field deployment. These coordinated material-mechanism-application innovations effectively overcome conventional limitations in sensitivity and multi-analyte detection [38].

A representative example of this integrated approach is the work by Shi et al., who developed a high-porosity gold (HPG)-based impedimetric aptasensor for the sensitive and selective detection of acetamiprid in fruits and vegetables. The HPG substrate was fabricated using electrochemical deposition, with optimized processing parameters yielding a sensing interface featuring an ultra-large specific surface area and abundant active sites. Experimental results demonstrated excellent selectivity and sensitivity toward acetamiprid, achieving a detection limit of 0.1 ppb, significantly lower than conventional methods. In real-sample testing, recovery rates remained stable between 90 and 105% with relative standard deviations below 5%, confirming strong reproducibility and reliability. Key innovations include: leveraging HPG’s unique porous structure to substantially increase aptamer loading capacity, thus amplifying signal response; enabling label-free detection through impedance spectroscopy analysis to streamline operations; and incorporating miniaturized circuitry to pioneer new technical pathways for field-deployable rapid detection. This research provides a simple, rapid, and sensitive new detection strategy for pesticide residue monitoring [77].

Beyond electrode-based systems, DNA aptamer-crosslinked hydrogel sensors represent another innovative approach for food safety monitoring, combining high specificity with intrinsic signal amplification. In these sensors, DNA aptamers serve dual roles—as selective recognition elements for target analytes and as crosslinking agents that drive the formation of three-dimensional hydrogel networks. When targets (e.g., antibiotics, toxins) bind to the aptamers, significant changes occur in hydrogel swelling or mechanical properties. Notably, Zhao et al. innovatively integrated optical/electrochemical signal transduction systems into the hydrogel matrix, achieving high-sensitivity detection of contaminants like chloramphenicol and ochratoxin A with limits of detection 0.01–0.1 ng/mL, representing a 10–100-fold improvement over traditional ELISA methods. The technology features rapid response (<15 min) and strong resistance to matrix interference, having been successfully applied to complex food matrices including milk and honey. Its modular design further allows expansion to other hazardous substance detection through interchangeable aptamer sequences [78].

These attributes collectively underscore a broader trend in aptasensor development: a critical transition from laboratory-scale proof-of-concept studies toward real-world deployment. Current research and engineering efforts are increasingly focused on miniaturization, integration with portable electronics, and intelligent data processing—key enablers for on-site, user-friendly, and automated monitoring systems. Technically, researchers have successfully reduced traditional detection setups to portable device scales through synergistic integration of microfluidic systems and microelectromechanical technologies, simultaneously cutting energy consumption by over 90%. More significantly, by incorporating machine learning algorithms and wireless transmission modules, next-generation sensors now enable real-time data analysis and remote monitoring, with convolutional neural network-based recognition systems achieving over 98% accuracy. Regarding application expansion, breakthroughs in wearable detection patches now enable continuous field monitoring while maintaining excellent sensitivity at 0.1 ppb levels. This technological evolution demonstrates how intelligent design enhancements are transforming theoretical concepts into deployable monitoring solutions.

#### 2.3.2. Enzyme Sensor

Enzyme sensors are biosensing technologies that detect pesticides through the specific interaction between immobilized enzymes and target analytes. Based on reaction mechanisms, they are primarily categorized into acetylcholinesterase (AChE) sensors and oxidase sensors. AChE sensors operate by measuring electrochemical or optical signal changes resulting from the inhibition of AChE activity by organophosphates and carbamates, offering rapid response and portability. Oxidase sensors, employing immobilized oxidases (e.g., tyrosinase) for pesticide recognition coupled with signal transduction, demonstrate higher sensitivity. Both types share advantages including real-time detection, simplified operation (minimal sample pretreatment), and field applicability, with AChE sensors already widely adopted for rapid agricultural product screening. However, AChE sensors are susceptible to pH/temperature interference, while oxidase sensors require improved specificity for certain pesticide classes, challenges partially addressed by sensor array integration. Current advancements in nanomaterial modification and microfluidic technology are driving enhanced performance in this field.

##### AChE Sensors

AChE sensors enable rapid screening by targeting the inhibitory effect of organophosphate and carbamate pesticides on AChE activity [79]. The core principle relies on pesticides irreversibly occupying the enzyme’s active sites, thereby hindering its catalytic hydrolysis of substrates (e.g., thioacetylcholine) into chromogenic products, resulting in diminished colorimetric signals. Quantifying the correlation between absorbance changes and inhibition rates allows effective assessment of pesticide residues. These sensors offer operational simplicity and low cost, making them particularly suitable for on-site rapid detection. However, limitations include insufficient sensitivity toward weakly inhibiting pesticides (e.g., chlorpyrifos) and susceptibility to interference from complex sample matrices, often necessitating complementary methods for result verification [80].

Nonetheless, significant progress has been made to address these challenges. For example, Kovarik et al. have recently demonstrated innovative strategies that enhance target capture efficiency and improve signal discrimination in complex environments, paving the way for next-generation aptasensors with expanded analytical capabilities. This platform enabled high-sensitivity real-time monitoring, precisely quantifying pesticide inhibition kinetics and demonstrating superior performance to conventional biochemical methods. Their research elucidated the specific binding mechanisms between pesticides and enzyme active sites, rapidly assessed the neurotoxic potency of different pesticides, and successfully screened the reactivation efficacy of antidotes. This technology provides a highly efficient and reliable platform for pesticide toxicity assessment and antidote development [81].

Technological innovation is further evident in the development of multimodal detection systems. Zhou et al., for example, integrated enzyme inhibition kinetics with electrochemical impedance spectroscopy analysis (using PCA-SVM algorithms achieving >90% classification accuracy) to successfully achieve simultaneous identification of avermectin, phoxim, dimethoate. Portable detection devices, such as paper-based chips costing less than $0.14, combined with impedance analysis techniques, enable on-site detection within 15 min [82]. Notably, Chen et al. established an AChE-regulated upconversion nanoparticle (UCNPs)-Cu^2+^ fluorescence system. Leveraging enzyme-activity-modulated fluorescence quenching, they achieved ultrasensitive detection of diazinon at 0.5 ng/mL, with results showing excellent consistency with GC-MS methods in real-sample analysis [83]. Advancements in enzyme immobilization processes and mobile terminal integration are now advancing pesticide detection technology toward cost-effectiveness, high throughput, and point-of-care applications.

##### Oxidase Sensor

Recent technological advances in pesticide residue detection have driven the development of highly sensitive analytical methods based on novel oxidases and their biomimetic analogs. These systems represent a significant evolution beyond conventional acetylcholinesterase (AChE)-based assays, which are often limited by poor environmental stability, susceptibility to pH and temperature fluctuations, and nonspecific inhibition. In contrast, the new generation of biomimetic materials offers marked improvements in detection specificity, operational stability under diverse conditions, and signal sensitivity—enabling more reliable and robust monitoring of pesticide contamination in food and environmental samples [84,85].

**Figure 5:**
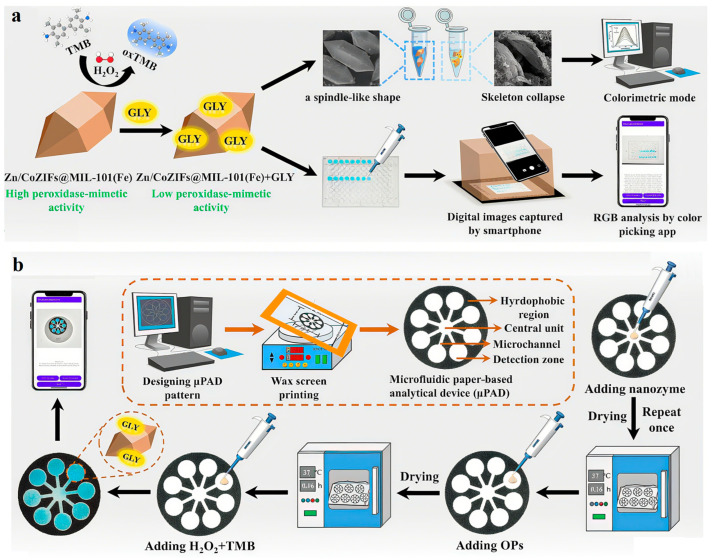
(**a**) Colorimetric and smartphone sensing platforms for glyphosate detection; (**b**) preparation of paper-based microfluidic analytical device [89].

This leap in performance is largely attributable to breakthroughs in the design of enzyme mimetics, where rational material engineering enables precise control over catalytic activity. A compelling example is the work of Mao et al., who developed porphyrin-based covalent organic frameworks (COFs) as functional nanozymes. These materials efficiently catalyze chromogenic reactions of substrates, achieving nanomolar detection limits for organophosphorus and carbamate pesticides [86]. The integration of advanced nanomaterial engineering with microfluidic platforms has led to substantial improvements in the performance of pesticide detection systems, particularly in terms of sensitivity and response speed. By leveraging nanostructure design and fluidic control, such systems now achieve detection sensitivities in the range of 0.01–0.1 μg/L and response times under 10 min—critical for rapid, on-site monitoring. A representative advancement in this direction is the work of Pang et al., who developed a bifunctional zirconia@zeolitic imidazolate framework-90 (ZrO_2_@ZIF-90) nanozyme for the simultaneous catalytic degradation and electrochemical detection of methyl parathion (MP). This nanozyme combines the inherent phosphatase-like hydrolytic activity of ZIF-90 with the catalytic enhancement provided by ZrO_2_, which is rich in Lewis acidic Zr(IV) sites. These sites promote the hydrolysis of MP into p-nitrophenol (p-NP), thereby enabling both target degradation and signal generation through the electroactive nature of the reaction product. The resulting catalytic efficiency of the ZrO_2_@ZIF-90 nanozyme reaches 3.2 × 10^4^ M^−1^s^−1^, among the highest reported for enzyme-mimicking systems. Under optimized conditions, this translates to a low detection limit of 0.53 μmol/L for MP [87], demonstrating the practical potential of rationally designed nanozymes in environmental and food safety applications. To augment specificity, researchers have innovatively combined aptamer recognition elements/molecularly imprinted polymers with nanozymes, markedly improving target identification in complex sample matrices. Technologically, the implementation of colorimetric-fluorescence dual-signal strategies effectively reduces false positive rates, while the development of paper-based sensors enables field-deployable rapid detection [88]. A compelling example of this dual progress is the work of Zhang et al., who developed a paper-based colorimetric sensing platform using a ternary metal–organic framework (MOF) composite, ZnCo-ZIFs@MIL-101(Fe). The synergistic electronic interaction among the Fe^3+^, Co^2+^, and Zn^2+^ metal centers—referred to as electronic hybridization—enhances charge transfer and catalytic activity, significantly boosting the peroxidase-like performance of the composite. This high catalytic efficiency enables the sensitive oxidation of chromogenic substrates in the presence of glyphosate, forming a visible color change that facilitates rapid visual or smartphone-assisted detection. The sensor demonstrates a wide linear detection range of 0.02–40 μg/mL for glyphosate, with an exceptionally low detection limit of 1 ng/mL [89], underscoring its potential for ultrasensitive, cost-effective, and field-deployable monitoring of pesticide residues in real-world settings (Figure 5).

The integration of intelligent sensing technologies has catalyzed a paradigm shift in detection systems. Researchers engineered a dual-layer electrode architecture that innovatively combines enzyme inhibition reactions with temperature regulation. Coupled with time-series impedance analysis and machine learning algorithms, this system achieves high-throughput detection of 32 samples within 15 min. It demonstrates 96.3% identification accuracy for common organophosphorus pesticides and a concentration prediction coefficient of determination (R^2^) of 0.953, with results showing strong concordance with conventional chromatography methods. This cost-effective, high-efficiency solution provides a robust tool for agricultural product safety monitoring [90].

Despite significant advancements, persistent challenges remain in enzymatic environmental stability and multi-residue simultaneous detection. Future research should prioritize developing stimuli-responsive materials (e.g., pH/temperature-sensitive covalent organic frameworks) and integrating miniaturized detection platforms to enhance practical utility.

## 3. Emerging Technologies and Trends

### 3.1. Nanotechnology

The field of modern pesticide residue detection is undergoing a technological revolution driven by nanotechnology. Nanostructures with unique physicochemical properties significantly enhance detection system performance through multiple synergistic mechanisms. Among various nanomaterials, semiconductor quantum dots have gained prominence due to their tunable fluorescence characteristics and flexible surface modification capabilities. These properties enable the establishment of specific interactions with target pesticide molecules, thereby amplifying optical signals and achieving ultra-trace detection [52]. Concurrently, noble metal nanoparticles (such as gold and silver) serve as ideal enhancement substrates for SERS through localized surface plasmon resonance phenomena. A practical application of this principle is demonstrated by Huang et al., who developed a flexible and eco-friendly SERS-active substrate composed of chitosan-stabilized silver nanoparticles on filter paper (Ch/AgNPs/paper) for the detection of chlorpyrifos residues in wheat samples. The chitosan matrix not only facilitates uniform dispersion and stabilization of AgNPs but also promotes analyte enrichment on the substrate surface, thereby improving both sensitivity and reproducibility. As a result, the Ch/AgNPs/paper platform enables clear enhancement of the characteristic Raman fingerprint of chlorpyrifos at trace concentrations. The method achieves reliable detection with a maximum tested concentration of 0.000558 mg/L (558 ng/L), demonstrating its high sensitivity and applicability for monitoring ultra-low levels of pesticide residues in complex food matrices [91]. Particularly noteworthy are silver-shell-coated star-shaped gold nanocomposite structures (AuNS@Ag), which not only effectively overcome matrix interference in complex samples but also achieve efficient target separation and signal amplification when coupled with magnetic Fe_3_O_4_@AuNP substrates [92].

#### 3.1.1. Innovative Applications of Quantum Dot Fluorescent Probes

Current research on quantum dot fluorescent probes primarily focuses on two technological pathways: quantum dot fluorescent probe systems and nanozyme-SERS integrated detection platforms. Bibliometric analysis reveals that over the past five years, quantum dot technology has achieved significant breakthroughs in detecting multiple pesticide residues due to its exceptional photophysical properties, including high quantum yield, narrow emission bandwidth, and tunable wavelengths, along with facile surface functionalization. Specifically, it demonstrates superior performance in detecting organophosphates, carbamates, and tetracyclines. Compared with traditional chromatography, quantum dot fluorescence sensing systems exhibit three distinct advantages: rapid response, cost-effectiveness, and field-deployable convenience [93].

Exemplifying recent progress in optical sensing, Xu et al. developed a ratiometric fluorescent probe by integrating green-emitting carbon dots (CDs) with red-emitting CdTe quantum dots (QDs) for the highly selective and quantitative detection of methyl parathion (MP). In this dual-emission system, the CdTe QDs serve as a stable reference signal, while the green fluorescence of the CDs acts as the responsive signal. Under alkaline conditions, MP undergoes rapid hydrolysis to produce p-nitrophenol (p-NP), which forms strong hydrogen bonds with the surface functional groups of the carbon dots. This interaction facilitates an efficient inner filter effect (IFE), selectively quenching the green fluorescence of the CDs. As a result, the overall emission color shifts visibly from green to red with increasing MP concentration, enabling both ratiometric quantification and naked-eye detection. The probe achieves a highly sensitive detection limit of 8.9 nM and exhibits a linear response across the concentration range of 0–100 μM. Notably, this IFE-based sensing mechanism, triggered by the enzymatic-like hydrolysis of MP, delivers exceptional analytical performance. Validation in real-world samples—including agricultural products and water—revealed excellent recovery rates ranging from 98.9% to 105.0%, confirming the probe’s accuracy, reliability, and applicability for environmental and food safety monitoring [94].

Regarding silicon quantum dot (SiQD)-based sensors, Xu et al. engineered amino-functionalized SiQDs that specifically bind with MP, inducing significant fluorescence quenching. This approach achieved a detection limit of 3.4 nM with outstanding anti-interference capability. Validation in real samples showed recovery rates of 96.5–103.2% and RSD < 4.5%, confirming its practical utility [95].

#### 3.1.2. Integrated Innovation of Multifunctional Nano-Detection Platforms

Research progress indicates that integrating SERS technology with aptamer functionalization strategies and microfluidic chip technology significantly enhances both the specificity and throughput of detection systems.

For instance, sensors fabricated via electrostatic layer-by-layer self-assembly using PDADMAC/PSS-modified gold@silver nanorods on filter paper substrates exhibit exceptional SERS enhancement performance and excellent signal consistency. These sensors achieve detection limits of 0.024 ng/cm^2^ for thiram and 0.018 ng/cm^2^ for methyl parathion, with the entire detection process requiring only 5 min and no complex pretreatment steps. Practical sample tests demonstrate recoveries ranging from 85% to 112% and relative standard deviations below 12%, validating the technique’s reliability [96].

Furthermore, the carbon quantum dot-aptamer composite sensing system developed by Guizhou University enables ultrasensitive detection of organophosphorus pesticides (e.g., dimethoate) at 0.1 μg/kg levels through precise modulation of silver nanoparticle fluorescence quenching effects. Remarkably, this system maintains robust stability in real-world samples such as lettuce and tomatoes. This technology has been successfully extended to detect multiple contaminants including paraquat and ethyl carbamate [97].

#### 3.1.3. Exploration of Emerging Detection Principles

Breakthroughs in quantum dot technology have been complemented by the distinct advantages demonstrated in metal-semiconductor heterostructure research. Self-powered photoelectrochemical sensors, exemplified by Z-scheme perovskite configurations, have achieved enhanced pesticide detection sensitivity through optimized band structure engineering, offering novel approaches for complex matrix analysis.

For instance, Huang et al. developed a self-powered PEC sensor based on a Z-scheme perovskite heterojunction for detecting profenofos pesticide in milk and cabbage. By constructing a CsPbBr_3_/Bi_2_S_3_ heterojunction material, they effectively promoted the separation of photogenerated charge carriers, significantly boosting the sensor’s photoelectric response. Experimental results indicated a good linear relationship within 0–100 μM, a remarkably low detection limit of 0.03 μM, and exceptional selectivity and stability. The team optimized the heterojunction’s band structure, enabling an external bias-free, self-powered detection mode that simplifies the process. This sensor demonstrated high accuracy and reliability in real-sample analysis, providing a novel portable method for monitoring pesticide residues in agricultural products with significant value for food safety applications [98].

Current challenges for nanomaterials in pesticide detection primarily focus on eliminating complex matrix interference and improving long-term stability. Future development directions include multiplex residue detection and integration with smartphone platforms. This technology offers a new, efficient solution for food safety monitoring [93].

### 3.2. Artificial Intelligence and Big Data

The field of modern pesticide residue detection is undergoing a paradigm shift driven by artificial intelligence and big data technologies. By integrating multisource sensing data with advanced computational models, detection systems have achieved a quantum leap in both accuracy and efficiency [99,100]. Current research is advancing along three primary technical pathways: intelligent spectral analysis systems, electronic sensory networks, and computer vision platforms, while forming a complementary technological matrix that leverages their respective advantages.

#### 3.2.1. Intelligent Spectral Analysis Technology

The field of spectral detection demonstrates a clear trend toward technological convergence, where the integration of hyperspectral and Raman spectroscopy with deep learning has emerged as the industry mainstream. In their latest study, Jiang et al. proposed a multiclassifier entropy weight method that innovatively combines Savitzky–Golay smoothing filters with Competitive Adaptive Reweighted Sampling dimensionality reduction. This approach enables nondestructive detection of pesticide residues in leafy vegetables. Its breakthrough lies in dynamically adjusting weight coefficients of base classifiers through entropy weighting, resulting in a fused model delivering 94.20% overall accuracy and a 0.93 Kappa coefficient, significantly outperforming traditional single classifiers and conventional fusion strategies. This advancement effectively resolves the industry challenge of high-dimensional spectral data redundancy, providing novel solutions for rapid screening of agricultural products with complex matrices [101].

**Figure 6:**
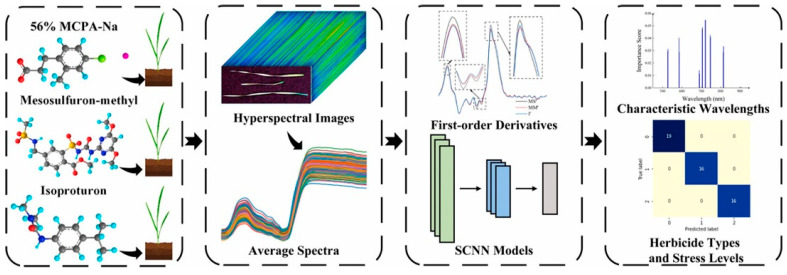
Hyperspectral imaging of shallow convolutional neural network (SCNN) for predicting early herbicides in wheat varieties [102].

The hyperspectral intelligent detection system developed by He et al. exhibits notable technical advantages. Operating within the 400–1000 nm spectral range, it employs a pioneering dual-model architecture: convolutional neural networks handle classification while partial least squares regression specializes in quantitative analysis, enabling single-sample detection within 30 s. Particularly noteworthy is its enhanced precision for complex samples like tea leaves through feature analysis of the red-edge band (690–760 nm). In practical applications within demonstration zones such as Taihu Lake Basin, the system incorporates XGBoost-based pest localization technology. This integration reduced pesticide usage by 40% while maintaining 92% control efficacy (Figure 6) [102].

Artificial intelligence holds broad application prospects in agriculture, yet still faces four major obstacles: First, the high deployment costs create barriers, as small-scale farmers struggle to afford intelligent sensors, drones, and other hardware and computing infrastructure. Second, the aging rural population lacks digital literacy, necessitating long-term training programs. Third, insufficient agricultural data accumulation, particularly the lack of localized crop growth data, affects the precision of model localization. Fourth, unstable network coverage in remote farmlands hinders real-time data transmission required for precision agriculture. Therefore, systemic solutions such as policy subsidies, digital skill enhancement for farmers, and rural digital infrastructure development are still needed to overcome these bottlenecks.

#### 3.2.2. Innovative Applications of Electronic Sensory Systems

In the field of non-spectral detection, electronic sensor systems demonstrate unique technological value. Recent research indicates that combined electronic nose and electronic tongue (E-tongue) systems integrated with machine learning algorithms can achieve efficient identification of pesticide residues in fruits [103]. By employing multivariate statistical methods such as principal component analysis (PCA) and linear discriminant analysis, along with classification models including decision trees and support vector machines, these systems can accurately distinguish pesticide residues across various fruits like strawberries and apples. Comparative studies reveal that electronic tongue systems exhibit superior performance in both classification accuracy and stability. Through multi-sensor data fusion combined with SVM/K-NN algorithms, e-tongue systems can precisely identify pesticide residues at different concentration levels.

#### 3.2.3. Algorithm Innovation and Engineering Optimization

Modern algorithms and engineering technologies are widely applied in the field of detection. From deep belief networks (DBN) to convolutional neural networks (CNN), and further to SERS technology combined with advanced materials, each method has, to varying degrees, enhanced the accuracy and efficiency of detection [104,105]. DBNs offer significant technical advantages and innovative potential in the detection of pesticide mixtures, particularly in handling the high-dimensional and complex spectral data generated by modern analytical instruments. Their primary strength lies in overcoming the limitations of manual and heuristic feature selection inherent in traditional spectral analysis. By leveraging multi-layer nonlinear transformations, DBNs enable adaptive dimensionality reduction and automatic extraction of discriminative features, making them highly effective for mining critical information from complex datasets [106]. This capability is exemplified in the work of Wu et al., who developed a hybrid DBN–support vector machine (SVM) model for the detection of fenvalerate and triazolone residues on lettuce leaves. In this approach, the DBN autonomously learns hierarchical representations of the spectral input, and the final layer of its restricted Boltzmann machines (RBMs) outputs a compact 100-dimensional feature vector. This dimensionality reduction not only simplifies the data structure but also preserves essential patterns for classification. By feeding these learned features into an SVM classifier, the hybrid model effectively mitigates the risks of underfitting and overfitting—common challenges when training deep models on limited datasets. The resulting DBN–SVM framework achieved high classification accuracy, with 98.89% on the training set and 95.0% on the independent test set, demonstrating that DBNs can robustly extract meaningful features from spectral data, and that their integration with classical classifiers further enhances predictive performance [107]. Complementing this work, CNNs have also shown great promise in quantitative pesticide analysis, particularly when combined with SERS. For instance, Li et al. developed a CNN-based method for the simultaneous quantification of thiram and pymetrozine in tea samples. The approach employed Au–Ag hollow cage nanostructures—synthesized using octahedral Cu_2_O as a sacrificial template—as highly sensitive SERS substrates. These results highlight the CNN’s superior ability to capture subtle, nonlinear spectral patterns associated with target analytes. Collectively, these studies demonstrate that deep learning models—whether DBNs for feature extraction and classification or CNNs for end-to-end regression—can significantly enhance the sensitivity, accuracy, and robustness of pesticide residue detection. Moreover, they provide a methodological blueprint for addressing analytical challenges in other complex industrial and environmental monitoring systems [108]. These studies establish an automated, high-precision, non-destructive testing paradigm for agricultural product safety, though future work should validate cross-scenario generalization through expanded sample diversity.

### 3.3. Development of On-Site Rapid Detection Technologies

In the domain of agricultural product quality and safety monitoring, rapid detection technologies tailored for non-laboratory environments are becoming increasingly critical. These portable analytical methods overcome the spatial and temporal constraints of conventional laboratory-based testing by enabling on-site, preliminary screening of pesticide residues within 10–30 min—thereby facilitating real-time, data-driven decisions in food safety regulation [109]. A notable advancement in this direction was reported by Xie et al., who developed a high-precision, rapid colorimetric sensing device based on a microfluidic chip integrated with a silicon photodetector for the detection of organophosphorus (OP) pesticide residues. To maximize assay efficiency, the microchannel geometry was optimized using computational fluid dynamics simulations, ensuring rapid and uniform mixing of reagents. The microfluidic chip was then fabricated using a self-developed mask-based liquid crystal display photocuring system, combining cost-effectiveness with high-resolution patterning. During operation, the device generates a measurable voltage signal proportional to the concentration of the target pesticide, establishing a robust linear calibration curve: y = 2.27 + 0.801x (where y is voltage output and x is concentration in mg·L^−1^), with a correlation coefficient (R^2^) of 0.985 and a LOD as low as 0.045 mg·L^−1^. Remarkably, the entire detection process is completed in just 60 s—substantially faster than both conventional lab methods and other field-deployable techniques. In addition to its speed, the system reduces sample consumption, enhances sensitivity, and ensures reliable quantification, all of which are essential attributes for practical on-site testing. Collectively, this integrated microfluidic-photodetection platform not only advances the state of the art in rapid pesticide screening but also provides a scalable and robust technical solution for real-world food safety surveillance, particularly in resource-limited or field-based settings [110].

#### Innovative Applications of Microfluidic Technology

Driven by the digital transformation of detection technologies, microfluidic systems have demonstrated unique value in pesticide residue analysis due to their miniaturization and high integration capabilities [111]. By consolidating multiple detection modalities, including electrochemical sensing, fluorescence labeling, SERS, and colorimetric analysis, onto a single microchip platform, this technology significantly enhances detection efficiency. Current microfluidic chip fabrication primarily employs three material systems with distinct advantages: cellulose-based substrates offering exceptional liquid self-propulsion, polydimethylsiloxane (PDMS) polymers delivering superior biocompatibility, and poly(methyl methacrylate) (PMMA) materials providing outstanding mechanical properties. Through precision micro-nanofabrication processes, these materials enable seamless integration of sample pretreatment and detection steps, establishing a robust technical foundation for next-generation portable detection devices.

Among these, paper-based microfluidics—leveraging the inherent wicking properties of cellulose—has emerged as a particularly promising platform for low-cost, field-deployable sensing. In this context, Yang et al. developed an innovative multilayer paper-based microfluidic device for the detection of organophosphorus (OP) pesticide residues. The device performs both qualitative and quantitative analysis by combining enzyme inhibition assays with an integrated resistive heating mechanism. A key innovation is the built-in heating layer, which actively maintains the reaction zone at an optimal temperature, thereby minimizing environmental variability and significantly improving the accuracy and reproducibility of enzymatic detection. Pesticide concentration is determined by measuring colorimetric changes in the detection zone using a spectrometer, enabling precise quantification. Under optimized conditions, the system exhibited a strong linear response to dichlorvos over the range of 0.05–0.5 mg/L (R^2^ > 0.99), with a limit of detection (LOD) as low as 0.0406 mg/L. Moreover, the method demonstrated high reproducibility, excellent specificity against common interferents, and long-term stability, confirming its reliability for real-world applications. These attributes collectively make the device well-suited for point-of-care and on-site monitoring of pesticide contamination in agricultural and environmental settings [112].

In microfluidic chip fabrication technology, researchers have developed a non-isothermal molding technique based on resistive heating principles for rapid production of PMMA microfluidic mixing chips. By optimizing current-controlled heating processes, this method significantly reduces energy consumption and thermal stress while maintaining high replication fidelity. The fabricated chips have been successfully implemented in portable pesticide residue detection systems, demonstrating exceptional analytical sensitivity (0.0375 mol^−1^). This innovative approach not only enhances manufacturing efficiency but also provides a cost-effective solution for pesticide screening applications [113].

Notably, although materials like PMMA demonstrate excellent performance in chip fabrication, PDMS retains dominance in practical applications due to its unique advantages. PDMS offers superior biocompatibility, optical transparency, and gas permeability, making it an ideal choice for microfluidic devices. The technology has been effectively deployed in environmental and food sample testing, achieving detection limits as low as 0.02 mg/L for organophosphorus pesticides. However, key technical challenges remain in achieving multiplex pesticide detection and overcoming interference from complex food matrices. Future research should prioritize developing self-calibration systems, enhancing anti-interference robustness, and deepening integration with mobile terminals like smartphones to advance practical implementation in agricultural product safety monitoring. Collectively, microfluidics provides a miniaturized, low-cost integrated solution for pesticide residue detection with significant developmental potential [114].

## 4. Comparison of Various Detection Methods

As shown in Table 1, current pesticide residue detection technologies are rapidly advancing toward higher sensitivity, faster response, and greater suitability for on-site applications. Various detection strategies have been developed, including spectroscopic methods (e.g., fluorescence, polarized spectroscopy, surface-enhanced Raman scattering, SERS), electrochemical aptamer sensors, colorimetric assays, and enzyme inhibition methods. In terms of technological innovation, Ma et al. proposed an ethanol dehydration-mediated isotropic contraction strategy (ED-Ag@PAM), which achieves uniform contraction by regulating water molecule exudation, significantly enhancing detection sensitivity (LOD down to 2.21 × 10^−12^ M) and signal homogeneity (RSD < 10%). This method is applicable to liquid environments and curved surface samples, demonstrating excellent versatility [30]. Fluorescence “off-on-off” strategies based on upconversion nanoparticles (UCNPs) reacting with copper ions or enzymes can significantly improve sensitivity; for instance, the LOD for diazinon can reach 0.05 ng/mL [82]. Regarding material design and performance optimization, different systems exhibit unique advantages. For example, Guo et al. constructed a dual-mode immunosensor using a double-layered 4-MBA-labeled AuMBA@AgMBA core–shell nanomaterial, where stable binding is achieved through dehydration condensation between antibody amino and carboxyl groups. The detection ranges for methamidophos and carbaryl were 0.3–2 μg/kg and 3–30 μg/kg, respectively, with recovery rates maintained between 82.78% and 115.12%, indicating strong quantitative capability [49]. Zhang et al. developed a MOFs hybrid sponge sensor using a fibrin membrane as a carrier to uniformly disperse Zr-LMOFs, achieving an LOD of 4.95 μg/L for methyl parathion and maintaining structural stability under organic solvents and acidic conditions [55]. In enzyme inhibition methods, Cao et al. utilized a genetically engineered bifunctional TrxA-PvCarE1 enzyme capable of simultaneously detecting organophosphorus pesticides and copper ions, with an LOD for dichlorvos as low as 2.4 × 10^−4^ mg/L, and batch reproducibility superior to traditional animal-derived acetylcholinesterase [84]. The advantages of biosensing include simple operation (e.g., paper-based sensors) [94] and low cost (e.g., enzyme-linked systems) [52], but it is susceptible to matrix interference (e.g., low sample recovery rates) [78]. Its applications cover fruits and vegetables [67], environmental water samples [57], and other fields, making it particularly suitable for on-site rapid screening.

In terms of convenience and application scenarios, multiple technologies are trending toward portability and intelligence. For instance, Qin et al. integrated a ratiometric fluorescent probe with a smartphone platform, enabling visual and quantitative detection of organophosphorus pesticides (LOD 16.3 nM) based on a Strecker-like reaction principle, using CdTe quantum dots as an internal standard and monitoring indole fluorescence changes. This approach is simple to operate and provides intuitive results [56]. Lin et al. designed an RB-Ag@Au nanoprobe utilizing the FRET mechanism, where the presence of organophosphorus pesticides induces nanoparticle aggregation, leading to fluorescence recovery with an LOD of 7.89 nM, and semi-quantitative analysis was achieved via a smartphone app [57]. In terms of sensitivity, SERS technology stands out (LOD 0.052 ng/cm^2^) [96], followed by fluorescence methods [94] and electrochemical methods (12.6 nM) [109]. Recovery rates generally fall within the 80–120% range, with data from HPLC-MS validation and silicon wafer simulation experiments showing recoveries of 92.7–109%, indicating high data reliability [85,96]. Regarding RSD, colorimetric methods typically exhibit values below 15% [85], while the signal variation coefficient of SERS substrates can be controlled within 10% [96]. Detection times range from seconds to several minutes [94,111]. In terms of portability, the nanozyme test strips developed by Luo et al. [97] and the integrated device by Pang et al. [111] are most suitable for on-site use, while the SERS test paper prepared by Chen et al. allows sampling through a simple “press-and-peel” operation, making it highly convenient [96]. Overall, nanomaterial-enhanced fluorescence methods offer superior sensitivity (LOD 0.01 ppb) [67], whereas electrochemical and enzymatic methods are more suitable for grassroots monitoring due to their lower cost and better portability. Future trends should focus on integrating nanosignal amplification with intelligent recognition elements [73] to construct multimodal sensing platforms that combine high sensitivity with on-site applicability.

## 5. Research Limitations and Future Directions

The pesticide residue detection field faces multifaceted technological hurdles. In chromatographic analysis, high-end instrumentation such as Ultra-Performance Liquid Chromatography-Quadrupole Time-of-Flight MS enables simultaneous screening of over 200 pesticides. However, its prohibitive acquisition cost and laborious sample pretreatment workflow severely limit widespread adoption. Regarding spectroscopic techniques, hyperspectral imaging systems preserve sample integrity but carry high price tags. Moreover, morphological variations across agricultural produce surfaces complicate spectral calibration. While SERS achieves sub-nanogram detection limits, its practical implementation is hampered by poor substrate reproducibility and spectral interference from plant pigments. Additionally, polarized spectroscopy and fluorescence detection methods exhibit extreme sensitivity to environmental conditions, necessitating stringent temperature and humidity control.

Technological advancements are poised to unfold through multidimensional breakthroughs. In functional materials development, composite structures integrating Molecularly Imprinted Polymers (MIPs) with MOFs demonstrate significant promise, potentially enhancing adsorption capacity by 5–10 times while mitigating environmental risks associated with nanomaterials. For integrated solutions, hybrid systems combining SERS fingerprint identification, microfluidic miniaturization (sample volume: <1 μL), and AI algorithms are emerging as transformative platforms. Within intelligent detection frameworks, low-cost edge-computing spectrometers coupled with cloud databases enable real-time field analysis, whereas fully automated sample processing robots reduce pretreatment time to under 10 min. Particularly noteworthy are breakthrough innovations in biomimetic olfactory sensors, which emulate insect chemoreception mechanisms. This approach holds potential to fundamentally overcome matrix adaptability challenges, thereby propelling detection technologies toward synergistic advancement in precision, intelligence, and accessibility.

## 6. Conclusions

Pesticide detection technology serves as a critical safeguard for food safety and ecological protection, with its advancement directly impacting the effectiveness of agricultural product quality supervision and sustainable agricultural development. From a food safety perspective, accurate pesticide residue detection effectively prevents substandard agricultural products from entering the market. On the environmental front, highly sensitive detection technologies can trace pesticide migration in soil and water at ppb levels, providing crucial data for ecological risk assessment. While significant progress has been made, current technologies still face many challenges: insufficient sensitivity, limited specificity, cumbersome procedures, and high costs. Future research should focus on developing an integrated “four-in-one” technological framework: enhancing specificity through biomimetic recognition materials, pushing sensitivity to femtogram levels via nano-enhancement effects (e.g., plasmon resonance), achieving streamlined “sample-to-result” operation with microfluidic chips, and reducing equipment costs through smart manufacturing. Only by achieving breakthroughs in both detection performance and practicality can we establish a comprehensive safety net spanning from farm to table.

## Figures and Tables

**Table 1:** Detection performance comparison of various detection methods.

Detection Scheme	Data Analysis Methods	Pesticides	Recovery Rate (%)	RSD (%)	LOD	Detection Time	Reference
SERS	Portable Raman spectrometer (785 nm), ImageJ (Bethesda, Maryland, USA) for nanoparticle sizing	Thiram, Thiabendazole (TBZ)	82.78–115.35	<10	Thiram: 5.26 × 10^−10^ g/mL; TBZ: 3.00 × 10^−8^ g/mL	12 min (dehydration) + 2 min (stand)	[30]
SERS with self-cleaning flexible sensor	CNN, CARS-PLS algorithms (R^2^ = 0.9963 for CNN)	Thiram	88.32–111.80	2.92–4.91	0.020 mg/L	<10 min	[31]
Microneedle (MN) patch SERS	Confocal Raman spectrometer (633 nm)	Thiram, TBZ	-	-	Thiram: 10^−7^ M; TBZ: 10^−8^ M	3 min	[35]
SERS-based immunoassay	Portable Raman spectrometer (785 nm), HALCON software (version:17.12, MVTec, München, Germany) for grayscale analysis	Acetamiprid, Carbendazim	82.78–115.35	<9	Acetamiprid: 0.27 μg/kg; Carbendazim: 1.71 μg/kg	-	[49]
Fluorescent sensors	FRET, PET, IFE, AIE mechanisms, Stern-Volmer equation, lifetime measurements	OPs, carbamates, organochlorines, pyrethroids	83.93–108.16	1.04–9	Glyphosate: 0.000207 nM; Thiram: 7 nM	Seconds to minutes	[52]
Fluorescent MOF Sponge	Fluorescence spectroscopy	Methyl parathion	90.1–107.5	≤5.9	4.95 ppb	10 min	[55]
Ratiometric Fluorescent Probe	Smartphone RGB analysis	Carbamates (MTMC)	90.1–107.5	≤5.9	18.6 nM	8 min	[56]
”Light Up” Fluorescent Sensor	Smartphone color recognition	Organophosphorus (OPs)	89.4–110.5	≤6.2	7.89 nM	20 min	[57]
Upconversion FRET Immunosensor	Fluorescence resonance energy transfer	Fipronil	95.95–137.07	≤5.9	0.01 ppb	30 min	[67]
Peroxidase-Mimicking Chemosensor	Differential pulse voltammetry (DPV)	Dimethoate	89.4–110.5	≤6.2	4.7 ppb	30 min	[70]
Photoelectrochemical Aptasensor	Photocurrent response	Carbendazim (CBZ)	98.93–106.10	≤5.9	0.33 pM	50 min	[72]
Electrochemical Aptasensor	DPV/CV	Imidacloprid, Thiamethoxam, Clothianidin	76.0–118.7	≤8.16	6.3–7.1 pg/mL	30 min	[73]
Impedimetric Aptasensor	Electrochemical impedance spectroscopy	Acetamiprid	92.0–107.5	≤6.1	0.34 nM	40 min	[76]
Upconversion fluorescence	Fluorescence resonance energy transfer (FRET), enzyme inhibition	Dimethoate	78.8–105.9	2.5–9.5	0.008 ng/mL	90 min	[78]
AChE-modulated fluorescence	Fluorescence “off-on-off” strategy	Diazinon	84.3–105.9	2.5–9.5	0.05 ng/mL	61 min	[82]
Bifunctional enzyme sensor	Fluorescence (IDA hydrolysis) and colorimetry (DTNB reduction)	10 OPs (e.g., dichlorvos, paraoxon) + copper compounds	80.08–112.38	0.6–15.99	0.00024 mg/L	30–60 min	[84]
COF-based colorimetry	Oxidase-mimetic catalysis, superoxide radical/^1^O_2_ generation	Fipronil, chlorfenapyr, flufenoxuron, etc.	80.0–82.4	-	2.7 ng/mL	30 min	[85]
Ratiometric fluorescence probe	Smartphone RGB analysis, internal filter effect (IFE)	Methyl parathion (MP)	98.9–105.0	≤4.5	8.9 nM	<2 s	[94]
SERS (Surface-Enhanced Raman Spectroscopy)	Portable Raman spectrometer (785 nm), UV-Vis, XRD, TEM/SEM	Methyl-parathion, thiram, chlorpyrifos	81.77–126.80	8.58–9.29	0.051 ng/cm^2^ (thiram)	<10 min	[96]
Colorimetric nanozyme sheet	Smartphone imaging, UV-Vis spectrophotometry	Glyphosate	89.0–96.1	1.89–5.38	0.175 mg/kg	10 min	[97]
Paper-based SERS	“Drop-wipe-measure” method, Raman mapping	Imidacloprid, ferbam	83.2–125	-	4.1 × 10^−6^ μg/mL	<5 min	[109]
Microfluidic mixer + Luminol	Photodetector voltage response	Quinalphos	-	-	0.035 mg/L	30 s	[111]

## Data Availability

Not applicable.
